# Supplementary material for: The Gut Microbiome in Depression and Potential Benefit of Prebiotics, Probiotics and Synbiotics: A Systematic Review of Clinical Trials and Observational Studies
Source: Int J Mol Sci. 2022 Apr 19;23(9):4494. doi: 10.3390/ijms23094494 (PMC9101152; doi:10.3390/ijms23094494)
Supplement: Supplementary file 1 [file ijms-23-04494-s001.zip › ijms-1639751-supplementary.pdf]

Table S1. Detailed taxa abundance changes in patients with major depressive disorder relative to healthy controls

Phylum	Class	Order	Family	Genus
Acidobacteria	Acidobacteria	Acidobacteriales	*Acidobacteriaceae*	*Chloracidobacterium*<br>↓Rong 2019
Actinobacteria<br>↑Chen 2018a, Chen 2018b, Zheng 2016, Chung 2019<br>↓Chen 2021	Actinobacteria	Actinomycetales<br>↔Zhang 2021	*Actinomycetaceae*<br>↑Chen 2018b, Zheng 2016, Chen 2020 (M-MDD)<br>↔Zhang 2021	*Actinomyces*<br>↑Chen 2020 (M-MDD)<br>↓Chen 2021<br>↔Zhang 2021
				Arcanobacterium<br>↑Rong 2019
				Mobiluncus<br>↑Chen 2021, Rong 2019
			Nocardiaceae<br>↑Chen 2018b	*Nocardia*<br>↑Rong 2019
			Streptomycetaceae<br>↑Chen 2018b	
		Bifidonbacteriales	*Bifidobacteriaceae*<br>↑Chen 2018b, Chung 2019, Rong 2019, Lai 2021	*Bifidobacterium*<br>↑Chung 2019, Yang 2020, Rong 2019, Lai 2021<br>↓Aizawa 2016, Liu 2016, Chen 2021
				Gardnerella<br>↑Rong 2019
		Corynebacteriales	*Tsukamurellaceae*	*Tsukamurella*<br>↑Rhee 2020
		Frankiales	*Frankiaceae*	*Frankia*<br>↓Rong 2019
		Micrococcales	*Micrococcineae*<br>↑Rong 2019	*Micrococcaceae*<br>↑Lai 2021

				Rothia<br>↑Rong 2019, Lai 2021
			Promicromonosporaceae	*Xylanimonas*<br>↑Rong 2019
		Mycobacteriales	*Corynebacteriaceae*<br>↑Chen 2021	*Uncultured*<br>↑Rhee 2020
		Streptosporangiales	*Thermomonosporaceae*	*Thermomonospora*<br>↓Rong 2019
	Coriobacteriia	Coriobacteriales<br>↑Zheng 2016<br>↓Chen 2021	*Atopobiaceae*<br>↑Lai 2021	*Atopobium*<br>↑Rong 2019, Lai 2021
				Olsenella<br>↑Rong 2019, Lai 2021
			Coriobacteriaceae<br>↑Zheng 2016, Chen 2020 (M-MDD), Rong 2019	*Collinsella*<br>↔Bai 2021
				Coriobacterium<br>↑Rong 2019, Lai 2021
			Unclassified<br>↓Chen 2021	
		Eggerthellales	*Eggerthellaceae*<br>↑Lai 2021	*Adlercreutzia*<br>↑Chung 2019<br>↓Yang 2020
				Eggerthella<br>↑Kelly 2016, Chung 2019, Chen 2020 (M-MDD), Rong 2019, Zhang 2021, Lai 2021
				Slackia<br>↑Rong 2019, Lai 2021

Bacteroidetes<br>↑Liu 2016, Chen 2021<br>↓Chen 2018a, Chen 2018b, Lin 2017, Zheng 2016, Chung 2019, Dong 2021	Bacteroidia<br>↑Liu 2020, Chen 2021	Bacteroidales<br>↑Liu 2020, Chen 2021	*Bacteroidaceae*<br>↑Chen 2020 (M-MDD), Zhang 2021<br>↓Lai 2021	*Bacteroides*<br>↑Liu 2016, Liu 2020, Yang 2020, Zhang 2021, Zheng 2020<br>↓Chen 2020 (M-MDD), Lai 2021
			Barnesiellaceae<br>↓Liu 2020	*Barnesiella*<br>↑Chen 2021<br>↓Liu 2016, Liu 2020
			Muribaculaceae	*Uncultured bacterium*<br>↓Liu 2020
			Odoribacteraceae	*Butyricimonas*<br>↓Liu 2016
				Butyricicoccus<br>↓Chen 2021
				Odoribacter<br>↓Liu 2016
			Porphyromonadaceae<br>↑Chung 2019, Zhang 2021<br>↔Chen 2018b	*Porphyromonas*<br>↓Rong 2019
				Parabacteroides<br>↑Chung 2019, Yang 2020, Chen 2021, Zhang 2021<br>↓Liu 2016<br>↔Bai 2021
			Prevotellaceae<br>↑Chen 2021, Chen 2020 (M-MDD)<br>↓Chen 2018b, Chung 2019, Zhang 2021	*Alloprevotella*<br>↑Zhang 2021<br>↓Zheng 2021

				Paraprevotella<br>↑Kelly 2016, Liu 2016, Zhang 2021
				Prevotella<br>↑Lin 2017, Rong 2019, Rhee 2020<br>↓Chung 2019, Zhang 2021
			Rikenellaceae<br>↑Zheng 2021, Zhang 2021<br>↓Zheng 2016<br>↔Chen 2018b	*Alistipes*<br>↑Chen 2021, Rhee 2020, Zheng 2021, Zhang 2021, Caso 2021<br>↓Zheng 2016, Liu 2016
			Tannerellaceae<br>↑Chen 2021	
			Unclassified/Uncultured<br>↑Yang 2020, Rhee 2020	
	Cytophagia	Cytophagales	*Cytophagaceae*<br>↓Lai 2021	
	Flavobacteriia	Flavobacteriales	*Flavobacteriaceae*<br>↓Lai 2021	
	Sphingobacteria	Sphingobacteriales or Chitinophagales	*Chitinophagaceae*<br>↓Chen 2018a, Chen 2018b, Rong 2019	
			Marniabilaceae<br>↓Chen 2018a, Chen 2018b	
			Saprospiraceae	*Saprospira*<br>↓Rong 2019
			Sphingobacteriaceae<br>↓Lai 2021	*Sphingobacterium*<br>↓Lai 2021
Chlorobi<br>↓Rong 2019	Chlorobia	Chlorobiales	*Chlorobiaceae*	*Chlorobium*

				Chloroherpeton<br>↓Rong 2019
Chlamydiae	Chlamydiae	Chlamydiales	*Chlamydiaceae*	*Chlamydophila*<br>↑Rong 2019
Firmicutes<br>↑Chen 2018b, Lin 2017, Chung 2019, Dong 2021<br>↓Liu 2020, Yang 2020, Chen 2021	Bacilli<br>↓Yang 2020	Bacillales	*Bacillaceae*	*Bacillus*<br>↑Rong 2019
			Unclassified	*Gemella*<br>↑Huang 2018
		Lactobacillales	*Aerococcaceae*	*Aerococcus*<br>↑Chen 2021
			Enterococcaceae<br>↑Lai 2021	*Enterococcus*<br>↑Liu 2020, Rong 2019, Lai 2021<br>↓Yang 2020
			Lactobacillaceae<br>↑Zheng 2016, Lai 2021	*Lactobacillus*<br>↑Rong 2019, Lai 2021<br>↓Aizawa 2016<br>↔Bai 2021
				Weissella<br>↓Zhang 2021
			Streptococcaceae<br>↑Zheng 2016, Chung 2019, Chen 2020 (M-MDD), Rong 2019	*Streptococcus*<br>↑Lin 2017, Chung 2019, Chen 2020 (M-MDD), Rong 2019, Lai 2021
				Lactococcus<br>↑Rong 2019

	Clostridia<br>↓Liu 2020, Chen 2021	Clostridiales<br>↑Zheng 2016<br>↓Liu 2020, Chen 2021	*Christensenallecae*<br>↓Liu 2020	*Christensenallecae R7 group*<br>↑Dong 2021<br>↓Liu 2020, Zheng 2020
			Clostridiaceae<br>↑Chen 2018b, Chen2018a<br>↓Huang 2018, Chen 2020 (Y-MDD), Zhang 2021	*Anaerotruncus*<br>↑Chen 2021, Zhang 2021
				Clostridium<br>↑Chen 2021, Rong 2019<br>↓Liu 2016, Yang 2020
				Clostridium sensu stricto<br>↓Chen 2020 (Y-MDD)
				Clostridium IV<br>↑Zheng 2016<br>↓Liu 2016
				Clostridium innocuum group<br>↔Bai 2021
				ClostridiumXIVa<br>↓Zheng 2016
				Clostridium XIX
				Clostridium XI<br>↑Lin 2017, Chung 2019<br>↓Liu 2016, Chen 2020 (Y-MDD)
				Clostridium XVIII<br>↑Chen 2020 (M-MDD)<br>↓Chen 2020 (Y-MDD)
				Hungatella<br>↑Chen 2021, Zhang 2021

			Clostridiales vadin BB60 group	*Uncultured organism*<br>↓Liu 2020
			Heliobacteriaceae<br>↑Rong 2019, Lai 2021	*Heliobacterium*<br>↑Rong 2019, Lai 2021
			Peptococcaceae<br>↑Lai 2021	*Desulfosporosinus*<br>↑Rong 2019
				Desulfitobacterium<br>↑Rong 2019, Lai 2021
			Unclassified	*Clostridiales bacterium S5-A14a*<br>↑Chen 2021
		Eubacteriales	*Eubacteriaceae*<br>↑Zheng 2016, Chen 2020 (M-MDD)	*Anaerovorax*<br>↓Chen 2020 (M-MDD)
				Eubacterium<br>↑Chen 2020 (M-MDD), Rong 2019<br>↓Liu 2020, Yang 2020, Chen 2021
				Eubacterium hallii<br>↓Chen 2021
				Eubacterium ventriosum<br>↓Liu 2020, Yang 2020<br>↔Bai 2021
			Intestinimonas	*Intestinimonas*<br>↑Zhang 2021
			Lachnospiraceae<br>↑Chen 2018a, Chen 2018b, Zheng 2016, Chung 2019, Chen 2020 (M-MDD)<br>↓Huang 2018, Chen 2021, Zheng 2021<br>↔Bai 2021	*Agathobacter*<br>↓Chen 2021

Anaerostipes<br>↑Zheng 2016, Chen 2020 (M-MDD)<br>↓Yang 2020, Chen 2021, Zheng 2021, Caso 2021
Coprococcus<br>↓Huang 2018, Liu 2016, Zheng 2016, Yang 2020, Chen 2021, Chen 2020 (M-MDD)
Dorea<br>↓Huang 2018, Yang 2020, Chen 2021
Eisenbergiella<br>↑Chen 2021<br>↔Zhang 2021
Fusicatenibacter<br>↑Dong 2021<br>↓Liu 2020, Chen 2021
GCA-900066575<br>↔Bai 2021
Lachnoclostridium<br>↑Chen 2021, Lai 2021<br>↔Bai 2021
Lachnospira<br>↔Bai 2021
Lachnospiracea group<br>↓Liu 2016, Chen 2021<br>↔Bai 2021
Roseburia<br>↑Chen 2020 (M-MDD)<br>↓Zheng 2016, Liu 2016, Chen 2021

		Sellimonas<br>↑Liu 2020
		Tyzzerella<br>↑Zhang 2021<br>↓Liu 2020, Chen 2021
		Unclassified<br>↔Bai 2021
	Peptoniphilaceae	*Finegoldia*<br>↑Chen 2021
	Peptostreptococcaceae<br>↑Chung 2019<br>↓Chen 2020 (Y-MDD), Zhang 2021	*Peptostreptococcus*<br>↑Huang 2018
	Oscillospiraceae<br>↑Lai 2021<br>↓Chen 2018b	*Fastidiosipila*<br>↑Chen 2021
		Filifactor<br>↑Rong 2019
		Oscillibacter<br>↑Rong 2019, Lai 2021<br>↓Liu 2016, Yang 2020, Chen 2020 (M-MDD)
		Ruminiclostridium<br>↓Chen 2021
	Ruminococcaceae<br>↑Chen 2018b, Zheng 2016, Rong 2019, Rhee 2020<br>↓Huang 2018, Liu 2020, Chen 2021, Chen 2020 (Y-MDD)	*Acetivibrio*<br>↓Liu 2016
		Anaerofilum<br>↑Kelly 2016

			CAG-352<br>↓Liu 2020
			CAG-56<br>↓Chen 2021<br>↔Bai 2021
			Eubacterium coprostanoligenes group<br>↓Liu 2020
			Faecalibacterium<br>↑Rhee 2020<br>↓Zheng 2016, Huang 2018, Liu 2016, Liu 2020, Yang 2020, Chen 2021, Chen 2020 (M-MDD), Dong 2021
			Flavonifractor<br>↑Liu 2020, Chen 2021, Zhang 2021
			Ruminococcus<br>↑Chung 2019<br>↓Liu 2016, Liu 2020, Yang 2020, Chen 2021
			Ruminococcus 2<br>↓Zheng 2020
			Ruminococcus gnavus<br>↑Zheng 2020
			Ruminococcus torques<br>↑Zheng 2020
			Subdoligranulum<br>↓Liu 2020, Yang 2020, Chen 2021
			Uncultured<br>↑Chen 2021

			Unclassified<br>↑Zheng 2016, Chen 2021	*Blautia*<br>↑Zheng 2016, Chung 2019<br>↓Huang 2018, Yang 2020, Chen 2021
				Parvimonas<br>↑Huang 2018, Zheng 2016
		Thermoanaerobacterales	*Thermoanaerobacteriaceae*	*Gelria*<br>↑Kelly 2016
		Unclassified<br>↑Chen 2021		
	Erysipelotrichia	Eerysipelotrichales	*Erysipelotrichaceae or Erysipelotrichaceae incertae sedis*<br>↑Chen 2018b, Zheng 2016<br>↓Chen 2021	*Bulleidia*<br>↑Huang 2018
				Coprobacillus<br>↑Chen 2021
				Faecalitalea<br>↑Zhang 2021<br>↔Bai 2021
				Holdemania<br>↑Kelly 2016, Chung 2019, Zhang 2021<br>↔Bai 2021
				Turicibacter<br>↑Kelly 2016, Chen 2021
	Negativicutes	Acidaminococcales	*Acidaminococcaceae*<br>↑Lai 2021<br>↓Zheng 2016, Chen 2020 (M-MDD)	*Acidaminococcus*<br>↑Rong 2019, Lai 2021
				Phascolarctobacterium<br>↑Yang 2020

				↓Zheng 2016, Chen 2020 (M-MDD)
		Selenomonadales ↑Rong 2019	*Selenomonadaceae*	*Megamonas* ↓Zheng 2016, Liu 2016, Chung 2019
				Mitsuokella ↓Liu 2016
				Selenomonas ↑Rong 2019
		Veillonellales	*Veillonellaceae* ↑Rong 2019, Lai 2021 ↓Zheng 2016	*Anaeroglobus* ↑Chen 2021
				Dialister ↑Rhee 2020 ↓Kelly 2016, Caso 2021
				Megasphaera ↑Rong 2019, Lai 2021
				Veillonella ↑Yang 2020, Chen 2020 (M-MDD)
	Mollicutes	Acholeplasmatales	*Acholeplasmataceae*	*Phytoplasma*
	Not assigned to family			*Colidextribacter* ↔Bai 2021
Fusobacteria ↑Chen 2021	Fusobacteriia ↑Chen 2021	Fusobacteriales ↑Chen 2021	*Fusobacteriaceae* ↑Chen 2021	*Fusobacterium* ↑Zheng 2020 ↓Liu 2016
			Leptotrichiaceae ↓Rong 2019	*Sebaldella* ↑Rong 2019

Proteobacteria ↑Chen 2021, Dong 2021 ↓Chen 2018b, Chung 2019	Alphaproteobacteria	Hyphomicrobiales	*Rhizobiaceae*	*Agrobacterium* ↓Rong 2019
			Methylobacteriaceae	*Methylobacterium* ↓Rong 2019
		Rhizobiales	*Hyphomicrobiaceae*	*Gemmiger* ↓Liu 2016
		Rhodospirillales ↓Liu 2020	*Rhodospirillaceae*	*Rhodospirillum* ↑Rong 2019
			Uncultured ↓Liu 2020	
	Betaproteobacteria ↑Chen 2021	Burkholderiales ↑Chen 2021	*Alcaligenaceae* ↓Chung 2019	*Alcaligenes* ↓Chen 2021
			Burkholderiaceae ↑Chen 2021	*Burkholderia* ↑Rong 2019 ↓Chen 2021
			Comamonadaceae	*Acidovorax* ↓Rhee 2020
				Comamonas ↓Liu 2016
			Oxalobacteraceae	*Oxalobacter* ↑Huang 2018
			Sutterellaceae ↓Chen 2018b, Chen 2018a, Zheng 2016, Chen 2020 (M-MDD)	*Parasutterella*
				Sutterella ↑Dong 2021 ↓Liu 2016, Chung 2019, Chen 2020 (M-MDD)

	Deltaproteobacteria	Bdellovibrionales	*Bdellovibrionaceae*	*Vampirovibrio*<br>↓Liu 2016
		Desulfovibrionales	*Desulfovibrionaceae*<br>↑Rong 2019	*Bilophila*<br>↑Caso 2021
				Desulfovibrio<br>↑Rong 2019<br>↓Liu 2020
				Lawsonia<br>↑Rong 2019
		Syntrophobacterales	*Desulfobaccaceae*	*Desulfobacca*<br>↓Rong 2019
	Epsilonproteobacteria	Campylobacterales	*Campylobacteraceae*<br>↑Chen 2021	*Campylobacter*<br>↑Chen 2021, Rong 2019
			Helicobacteraceae	*Helicobacter*<br>↓Rong 2019
	Gammaproteobacteria<br>↑Liu 2020, Chen 2021	Aeromondales<br>↓Zheng 2021	*Succinivibrionaceae*<br>↓Zheng 2021	*Succinivibrio*<br>↓Zheng 2021
		Alteromondales<br>↓Rong 2019	*Alteromonadaceae*	*Marinobacter*<br>↓Rong 2019
			Pseudoalteromonadaceae	*Pseudoalteromonas*<br>↓Rong 2019
		Enterobacterales<br>↑Chen 2021	*Enterobacteriaceae*<br>↑Chen 2021, Rong 2019<br>↓Chen 2018b	*Citrobacter*<br>↓Yang 2020, Zheng 2020
				Cronobacter<br>↑Rong 2019
				Enterobacter<br>↑Rong 2019

				Escherichia/Shigella<br>↑Chen 2021, Rong 2019, Rhee 2020, Zhang 2021<br>↓Liu 2016
				Klebsiella<br>↑Lin 2017, Chen 2021, Rong 2019<br>↓Yang 2020<br>↔Bai 2021
				Raoultella<br>↑Chen 2021
				Salmonella<br>↑Rong 2019
				Unclassified<br>↑Chen 2021
			Morganellaceae	*Morganella*<br>↑Chen 2021
			Yersiniaceae	*Yersinia*<br>↑Rong 2019
		Legionellales	*Coxiellaceae*	*Coxiella*<br>↑Rong 2019
		Pasteurellales<br>↓Zhang 2021	*Pasteurellaceae*<br>↓Rong 2019, Zhang 2021	*Aggregatibacter*<br>↓Rong 2019
				Haemophilus<br>↑Liu 2016<br>↓Rong 2019, Zhang 2021
				Rodentibacter<br>↑Chen 2021
		Pseudomonadales<br>↑Chen 2021	*Moraxellaceae*	*Moraxella*<br>↓Chen 2021

			Pseudomonadaceae	*Pseudomonas*<br>↑Huang 2018, Rong 2019<br>↓Rhee 2020
		Purple sulfur bacteria	*Chromatiaceae*	*Thiocystis*<br>↓Rong 2019
		Vibrionales	*Vibrionaceae*	*Vibrio*<br>↑Rong 2019
Spirochaetes	Spirochaetia	Xanthomonadales	*Xanthomonadaceae*	*Stenotrophomonas*<br>↑Rong 2019
		Spirochaetales	*Spirochaetaceae*	*Spirochaete*<br>↑Rong 2019
				Treponema<br>↑Rong 2019
Verrucomicrobia	Verrucomicrobiae		*Sphaerochaetaceae*	*Sphaerochaeta*<br>↑Rong 2019, Lai 2021
Uncultured		Verrucomicrobiales	*Akkermansiaceae*	*Akkermansia*<br>↑Rong 2019
				Candidatus_Saccharimonas<br>↓Chen 2021
				Candidatus_Soleaferrea<br>↔Bai 2021

Note. ↑, increased abundance in patients with major depressive disorder (MDD) relative to healthy controls (HC); ↓, decreased abundance in patients with MDD relative to HC, ↔, unspecified change in abundance in patients with MDD relative to HC; M-MDD, middle-aged MDD subgroup; Y-MDD, young MDD subgroup.
